# A preliminary study on the inorganic carbon sink function of mineral weathering during sediment transport in the Yangtze River mainstream

**DOI:** 10.1038/s41598-022-07780-6

**Published:** 2022-03-07

**Authors:** Xinbao Zhang, Jingcheng Luo, Xiaoguo Wang, Jialiang Tang, Tao Peng

**Affiliations:** 1 Key Laboratory of Mountain Environment Evolution and Regulation, Institute of Mountain Hazards and Environment, Chinese Academy of Sciences, Chengdu, 610041 China; 2 Faculty of Geosciences and Environmental Engineering, Southwest Jiaotong University, Chengdu, 610031 China; 3 Puding Karst Ecosystem Observation and Research Station, Chinese Academy of Sciences, Puding, 550081 China

**Keywords:** Carbon cycle, Biogeochemistry, Climate sciences, Hydrology

## Abstract

This study proposed that the dissolution of calcium and magnesium minerals in river sediment could sequester CO_2_ and function as a carbon sink. Based on the published study, "the contents and chemical and mineral compositions of the suspended particulate materials in the Yangtze River and their geological environmental implications” by Ding Tiping, the contents of CaO, MgO, calcite and dolomite in suspended sediment collected from 25 sampling points in the mainstream and 13 sampling points in the tributaries of the Yangtze River in 4 sampling campaigns during 2003–2007 were used to calculate the total inorganic carbon sink (TCS) capacity and nonsubstantial and substantial inorganic carbon sink (NSCS and SCS) capacities of suspended sediment along the river. Due to the reduction in the sediment yield, the TCS, NSCS and SCS of the Cuntan–Datong section during 2006–2019 decreased by 18.52 × 10^6^ tons, 12.24 × 10^6^ tons and 8.72 × 10^6^ tons, respectively, compared to the period before 2002. The average annual sedimentation of the Three Gorges Reservoir (TGR) was 114.5 × 10^6^ tons, and the related TCS and SCS losses were 6.76 × 10^6^ tons and 2.29 × 10^6^ tons, respectively, which were equivalent to 7.9 and 2.7 percent of the 85.8 × 10^6^ tons of CO_2_ emissions reduced by the clean energy production of the Three Gorges Hydropower Station. The TCS of global rivers was estimated as 757 × 10^6^ tons (the SCS was more than one quarter of the TCS), which is equivalent to 71.6% of the TCS by global rock weathering with 1.06 × 10^9^ tons of sequestered CO_2_. The collision and erosion of river sediment caused by turbulence in the processes of sediment transport (off-site rock weathering) could promote the dissolution of minerals. Therefore, it is reasonable that the dissolution rate of calcium and magnesium minerals for offsite rock weathering was much higher than that for in situ rock weathering.

## Introduction

The weathering of rocks containing silicate and carbonate minerals consumes CO_2_, which is an important inorganic carbon sink in the global carbon cycle^1^. An entire rock weathering process includes in situ weathering in the crust and off-site weathering during sediment transport. Atmospheric and aquatic CO_2_ is consumed by the weathering of silicate and carbonate minerals to form soluble bicarbonates in solution, which are transported into the sea through rivers. Some of the CO_2_ in the bicarbonates is deposited as Ca–MgCO_3_ (biological or chemical deposition) in the sea and is called the “Substantial Inorganic Carbon Sink”, and some of it is released back into the atmosphere and can be called the “Nonsubstantial Inorganic Carbon Sink”^2^. The chemical equations for Ca–Mg ions in calcium and magnesium silicates to consume and release CO_2_ during the weathering and deposition processes are expressed as follows:

1 $$\left( {{\text{Ca}}{-}{\text{Mg}}} \right)_{2} {\text{SiO}}_{4} + 4{\text{CO}}_{2} + 4{\text{H}}_{2} {\text{O}} \to \left( {{\text{Ca}}{-}{\text{Mg}}} \right)_{2} \left( {{\text{HCO}}_{3} } \right)_{4} + {\text{H}}_{4} {\text{SiO}}_{4}$$

2 $$\left( {{\text{Ca}}{-}{\text{Mg}}} \right)_{2} \left( {{\text{HCO}}_{3} } \right)_{4} \to 2\left( {{\text{Ca}}{-}{\text{Mg}}} \right){\text{CO}}_{3} + 2{\text{CO}}_{2} \uparrow + {\text{H}}_{4} {\text{SiO}}_{4}$$

In Eq. (2), the CO_2_ deposited via (Ca–Mg)CO_3_ is a substantial carbon sink (SCS), and the CO_2_ that is released is a nonsubstantial carbon sink (NSCS). In this manuscript, all the carbon sink terms refer to inorganic carbon sinks.

The chemical equation of absorbing and releasing CO_2_ by the Ca–Mg carbonate compounds during the weathering and deposition processes is expressed as follows.

3 $$\left( {{\text{Ca}}{-}{\text{Mg}}} \right){\text{CO}}_{3} + {\text{CO}}_{2} + {\text{H}}_{2} {\text{O}} \to \left( {{\text{Ca}}{-}{\text{Mg}}} \right)\left( {{\text{HCO}}_{3} } \right)_{2} \to \left( {{\text{Ca}}{-}{\text{Mg}}} \right){\text{CO}}_{3} \downarrow + {\text{CO}}_{2} \uparrow + {\text{H}}_{2} {\text{O}}$$

The CO_2_ consumed by carbonate weathering is a nonsubstantial carbon sink (NSCS), and it is released after entering the sea through rivers.

Suchet and Probst concluded that the CO_2_ consumption by rock weathering was mainly affected by the flow rate on the rock surface, atmospheric temperature and rock type^3^. They analyzed the data of surface runoff and major dissolved elements in 232 monolithologic basins in France and established a GEM-CO_2_ model to estimate the amount of atmospheric CO_2_ consumed by rock weathering based on the empirical relationships. The model was considered one of the main achievements of the international geological correlation program (IGCP) 404 project. Gaillardet et al. collected the hydrochemical data of 60 major rivers around the world and calculated that the global silicate weathering carbon sink amount was 1.4 × 10^8^ t C per year and the carbonate weathering carbon sink amount was 1.48 × 10^8^ t C per year through an inversion model^4,5^. It has been largely reported that calcium and magnesium minerals in sediment can dissolve CO_2_ and function as carbon sinks during river sediment transport, but the effects of human activities (such as damming) on the potential carbon sink capacity during sediment transport have not been reported until now^6–11^. Therefore, the objectives of this research are to (1) elucidate the mineral compositions and transport dynamics (offsite rock weathering) in the Yangtze River, (2) estimate the potential carbon sink capacity of suspended sediment transported in the Yangtze River, and (3) evaluate the effects of damming on the potential carbon sink capacity of suspended sediment.

## Materials and methods

**Figure 1:**
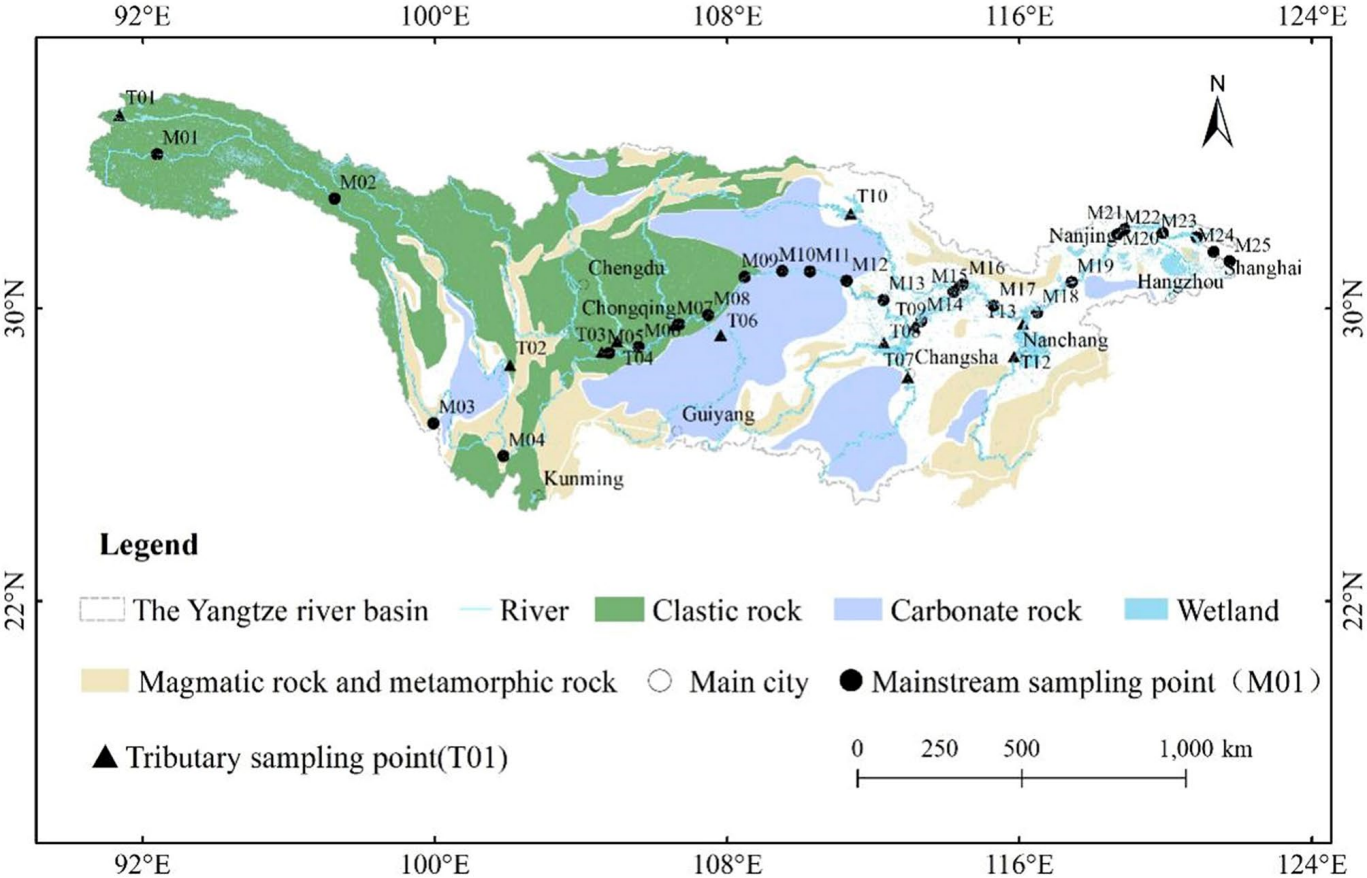
The map of Yangtze River Basin and the sampling points (Cited from Ding et al.^12^). *Note*: The sketch of geological map in the study region was redigitized from Ding’s publication using ARCGIS 10.4 (www.esri.com/en-us/store/overview).

**Figure 2:**
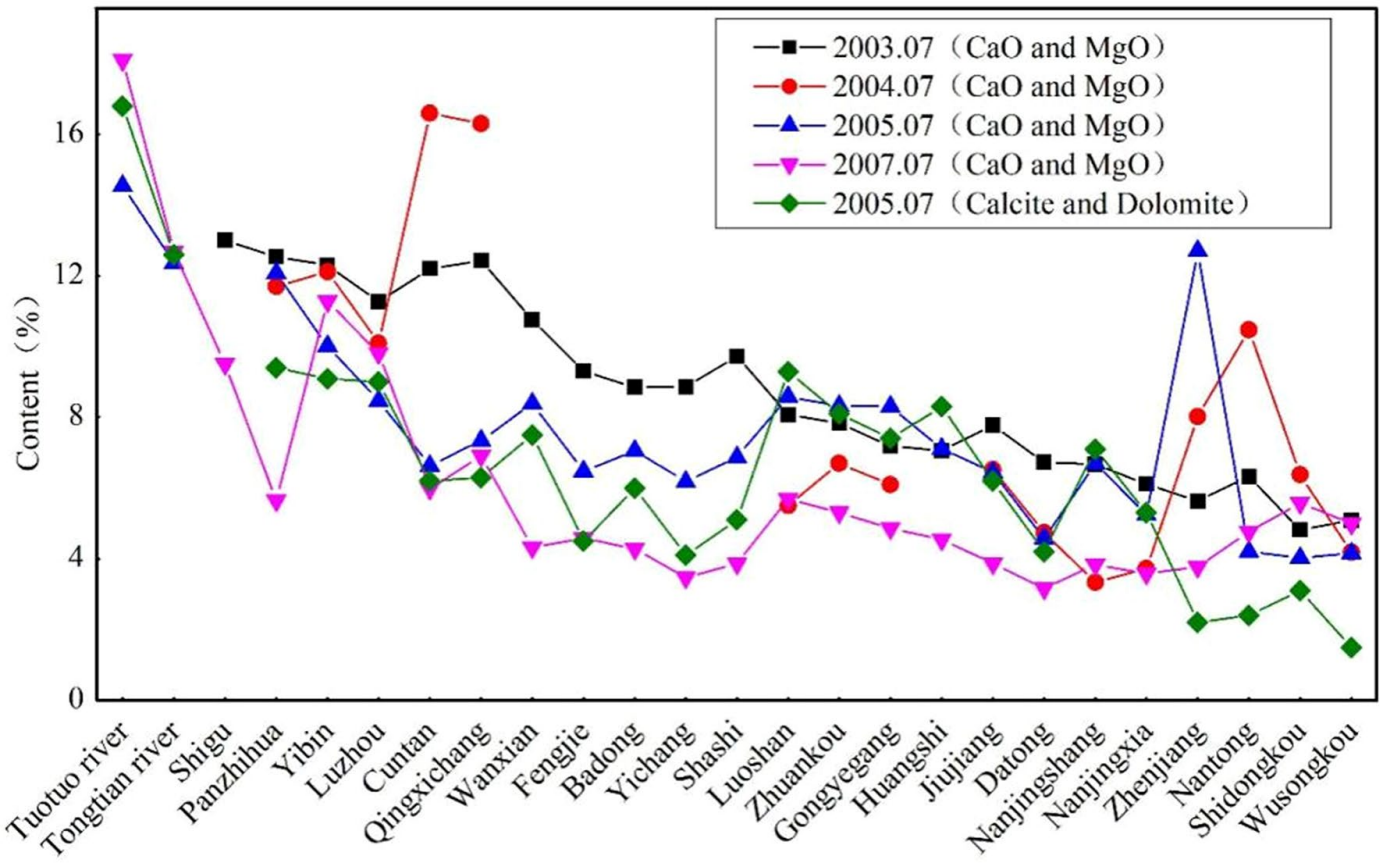
The variations of CaO, MgO, calcite and dolomite contents in suspended sediment along the mainstream of the Yangtze River.

This study analyzed the chemical element and mineral composition data of suspended sediments at 25 sites and 13 sampling points in tributaries from the upper reaches to the estuary of the Yangtze River from 2003 to 2007 according to Ding Tiping's publication^12^. Twenty-five sampling sites in the mainstream and 13 sites in the tributaries of the Yangtze River were sampled 4 times (July 2003, April 2004, July 2005 and July 2007) during 2003–2007, as shown in Fig. 1 and Table 1. The variations in the CaO, MgO (during 4 campaigns), calcite and dolomite contents (one campaign in July 2005) in suspended sediment of the Yangtze River along the river are shown in Fig. 2.

**Table 1:** Sampling points of suspended sediment in the main stream and tributaries of the Yangtze River.

Sampling point	Station name	River/lake	Location	Distance from source (km)
M01	Tuotuo river	Yangtze River mainstream	Under the Tuotuo River Bridge in Qinghai	130
M02	Tongtian river	Yangtze River mainstream	Yushu Zhimenda Observation Station	980
M03	Shigu	Yangtze River mainstream	Lijiang Shigu Observation Station	1820
M04	Panzhihua	Yangtze River mainstream	Panzhihua Observation Station	2754
M05	Yibin	Yangtze River mainstream	Yibin Observation Station	3480
M06	Luzhou	Yangtze River mainstream	Luzhou Observation Station	3580
M07	Cuntan	Yangtze River mainstream	Chongqing Cuntan Observation Station	3855
M08	Qingxichang	Yangtze River mainstream	Chongqing Qingxichang Observation Station	4020
M09	Wanxian	Yangtze River mainstream	Chongqing Wanzhou Observation Station	4200
M10	Fengjie	Yangtze River mainstream	Chongqing Fengjie Observation Station	4300
M11	Badong	Yangtze River mainstream	Badong Observation Station	4400
M12	Yichang	Yangtze River mainstream	Yichang Nanjinguan Observation Station	4515
M13	Shashi	Yangtze River mainstream	Shashi Brick and Tile Factory Observation Station	4620
M14	Luoshan	Yangtze River mainstream	Honghu Luoshan Observation Station	4850
M15	Zhuankou	Yangtze River mainstream	Wuhan Zhuankou Observation Station	5030
M16	Industrial Port	Yangtze River mainstream	Wuhan Industrial Port Observation Station	5157
M17	Huangshi	Yangtze River mainstream	Huangshi Observation Station	5260
M18	Jiujiang	Yangtze River mainstream	Jiujiang Observation Station	5426
M19	Datong	Yangtze River mainstream	Chizhou Datong Observation Station	5650
M20	Nanjingshang	Yangtze River mainstream	Nanjing Jiangning River Observation Station	5880
M21	Nanjingxia	Yangtze River mainstream	Nanjing Shili Changgou Observation Station	5950
M22	Zhenjiang	Yangtze River mainstream	Zhenjiang Observation Station	6020
M23	Nantong	Yangtze River mainstream	Nantong Observation Station	6130
M24	Shidongkou	Yangtze River mainstream	Shanghai Shidongkou Observation Station	6250
M25	Wusongkou	Yangtze River mainstream	Shanghai Wusongkou Observation Station 23 km	6300
T01	Chumar River	Chumar River	Under the Chumar River Bridge in Qinghai	430
T02	Mianning	Yalong River	Along the river in Yarlung, Mianning	2874
T03	Gaochang	Minjiang River	Yibin Gaochang Observation Station	3480
T04	Fushun	Tuojiang River	Fushun observation station	3580
T05	Linjiangmen	Jialing River	Chongqing Linjiangmen Observation Station	3850
T06	Wulong	Wujiang River	Chongqing Wulong Observation Station	4020
T07	Changsha	Xiangjiang River	Changsha Observation Station	4720
T08	Nanju	Dongting Lake	Nanju Observation Station	4720
T09	Chenglingji	Dongting Lake	Yueyang Chenglingji Observation Station	4720
T10	Danjiangkou	Danjiangkou Reservoir	Danjiangkou Reservoir Dam Observation Station	5157
T11	Jijiazui	Han River	Hankou Jijiazui Observation Station	5157
T12	Nanchang	Ganjiang River	Under Ganjiang Bridge in Nanchang	5465
T13	Xieshan	Poyang Lake	Jiujiang Xieshan 1 km south	5465

The suspended sediments were not measured at some sampling sites in April 2004 because the sediment concentration in water was too low to be collected in the dry season of April 2004. Furthermore, the sediment yields in the flood seasons of May–October account for more than 75% of the yield for a year; therefore, the mean values of the chemical and mineral contributions of the three campaigns in July were used for the following calculation. However, the value in July 2005 at Zhenjiang was irregular and eliminated from the calculation.

The carbon sink effects of silicate and carbonate mineral weathering during the sediment transport processes were estimated based on the variation in the CaO, MgO, calcite and dolomite contents in suspended sediments along the mainstream of the Yangtze River. In this study, we defined the CO_2_ consumed per unit weight by the dissolution of CaO + MgO in silicate and carbonate minerals of suspended sediment to bicarbonate forms as the total carbon sink (TCS) capacity of suspended sediment (C_1_, t/t). In more detail, the molar masses per unit mass of CaO, MgO, calcite (CaCO_3_) and dolomite (CaCO_3+_MgCO_3_) were calculated based on the contents of CaO, MgO, calcite and dolomite in the measured sediment samples at each site, and the respective carbon sink capacities (which consumed CO_2_ per ton during sediment transport) were calculated based on the same molar mass of Ca/Mg. According to the definition and Eq. (2) mentioned above, the CO_2_ consumed by the weathering of Ca/Mg carbonate minerals is a nonsubstantial carbon sink (NSCS C_c_); this yields approximately one mole of CO_2_ from one mole of weathered CaCO_3_, which is released again when carried into the oceans. Moreover, from Eq. (1), we infer that one mole of the CO_2_ consumed by the weathering of one mole Ca/Mg silicate minerals is a substantial carbon sink (SCS, C_s1_) and the other mole is a nonsubstantial carbon sink (NSCS, C_s2_):

4 $${\text{C}}_{1} = {\text{C}}_{2} + {\text{C}}_{3}$$

5 $${\text{C}}_{2} = {\text{Cc}}_{{\text{t}}} + {\text{C}}_{{{\text{s}}2}}$$

6 $${\text{C}}_{3} = {\text{C}}_{{{\text{s}}1}}$$

where C_1_ is the TCS capacity (t/t); C_2_ is the NSCS capacity (t/t); C_3_ is the SCS capacity (t/t); C_c_ is the NSCS capacity of Ca/Mg carbonate minerals (t/t); C_s1_ is the SCS capacity of calcium-magnesium silicate minerals (t/t); and C_s2_ is the NSCS capacity of Ca/Mg silicate minerals (t/t).

In fact, the SCS capacity was calculated by subtracting the NSCS capacity (based on the molar mass of sequestered CO_2_ by weathering of calcite and dolomite) from the TCS capacity (based on the molar mass of sequestered CO_2_ by weathering of all minerals containing CaO/MgO) in this study. Subsequently, the influence of sediment yield changes on the carbon sink contributed by off-site rock weathering in the Yangtze River was preliminarily evaluated via an “ideal mainstream segment” method, especially for comparing the two periods of preimpoundment (1956–2000) and postimpoundment (2003–2017) of the Three Gorges Reservoir (TGR), which was gradually impounded from 2003 to 2008.

## Results and discussions

### CaO, MgO, calcite and dolomite contents of suspended sediments in the Yangtze River and its main tributaries

Figure 2 shows a declining tendency of the CaO + MgO and calcite + dolomite of suspended sediments in the mainstream of the Yangtze River from upstream to downstream. The total CaO + MgO contents along the Yangtze River were as follows: Tuotuo River, 16.33%; Yibin, 11.43%; Cuntan, 10.35%; Yichang (below the Three Gorges Dam), 6.17%; Wuhan Industrial Port, 6.61%; Datong, 4.80%; and Wusongkou, 4.60%. The total calcite + dolomite contents also decreased along the river as follows: Tuotuo River, 16.8%; Yibin, 9.1%; Cuntan, 6.2%; Yichang, 4.1%; Wuhan Industrial Port, 7.4%; Datong, 4.2%; and Wusongkou, 1.5%. The three stations closest to the estuary were dominated by dolomite and free of calcite.

Due to the dramatic terrain changes in the upper reaches of the Yangtze River, strong gravity erosion and physical weathering, the CaO + MgO and calcite + dolomite contents were high in the mainstream section of the Jinsha River above Yibin and decreased from the headwater to the estuary, which clearly illustrated the dissolution of calcium-magnesium silicate and carbonate minerals during the process of sediment transport in the river. The declining rates of CaO + MgO and calcite + dolomite contents in the upper reaches of the Yangtze River above Yichang were 0.12%/100 km and 0.29%/100 km, respectively, while the rates below Yichang were 0.09%/100 km and 0.15%/100 km, respectively. The declining rate of calcite and dolomite contents was higher than that of CaO + MgO contents, which indicated that carbonate minerals were more likely to be dissolved than calcium-magnesium silicate minerals during sediment transport. Because the upstream reaches had greater slope gradients, faster flow velocities and consequently higher mineral dissolution rates, the CaO + MgO and calcite + dolomite contents of suspended sediments in the upper reaches had a higher declining rate than those in the middle and lower reaches.

The CaO + MgO and calcite + dolomite contents of the suspended sediment in the main tributaries of the Yangtze River were as follows: Minjiang River: 9.75% and 6.3%; Jialing River: 5.40% and 5.5%; Wujiang River: 10.87%; Xiangjiang River: 4.75%; Hanjiang River: 4.41%; and Ganjiang River: 2.87% (only CaO + MgO contents and no calcite + dolomite content data for the last four tributaries). Except for the Wujiang River, CaO + MgO contents and calcite + dolomite contents in the Minjiang River were higher than those in other tributaries and close to the Jinsha River (Yibin site) because the Minjiang River basin had similar environments for erosion and sediment transport to the Jinsha River Basin. The CaO + MgO contents of suspended sediments in the Wujiang River were quite different between July 2003 and July 2007, i.e., 6.67% and 15.06%, respectively. The relatively high contents might be due to its widespread distribution of carbonate rock.

### Carbon sink capacity during suspended sediment transport in the Yangtze River mainstream

**Figure 3:**
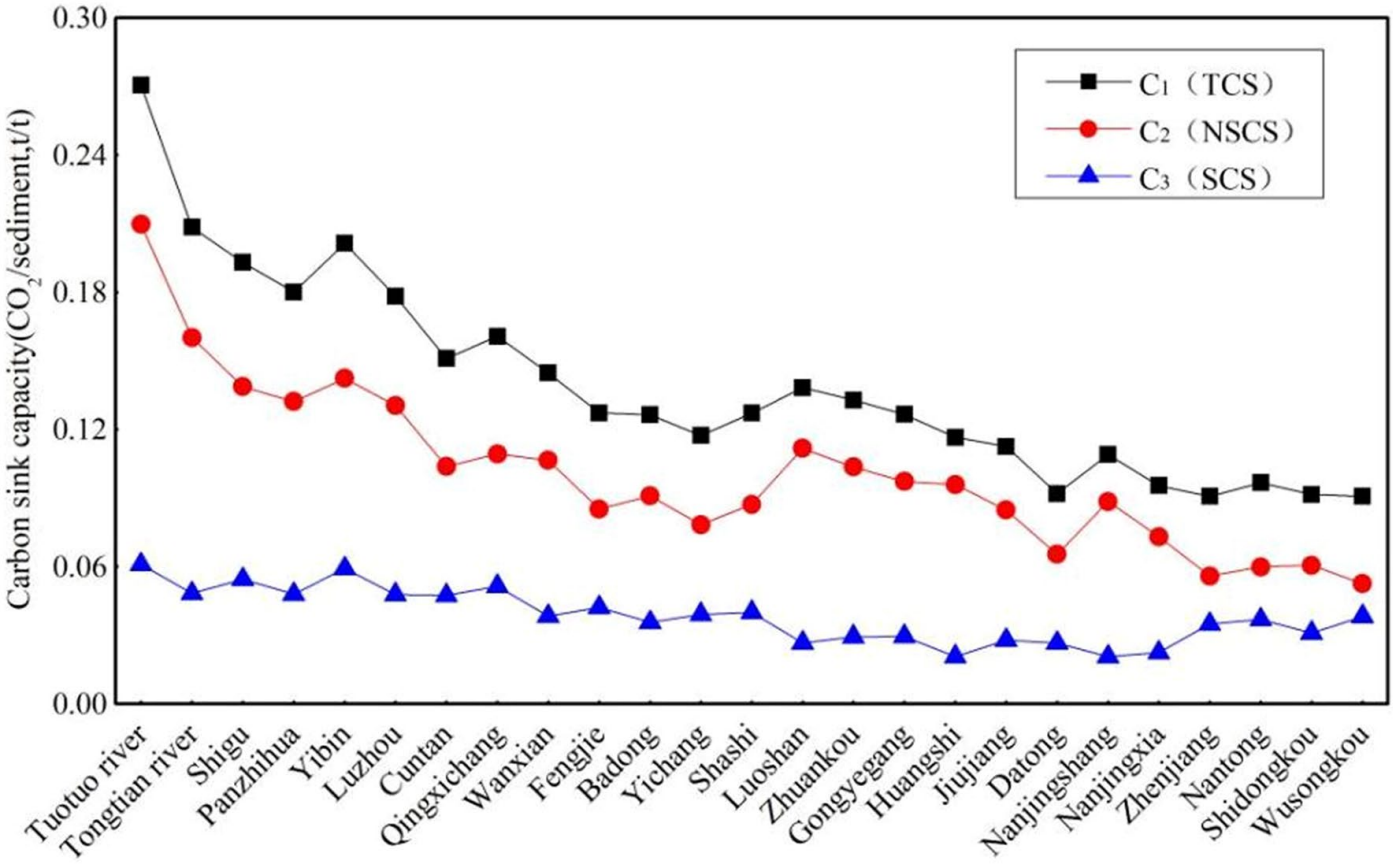
The variation of carbon sink capacities of suspended sediment in the Yangtze River.

According to Eqs. (1)–(6), the calculated TCS capacity (C_1_) decreased gradually from the headwater to the estuary (Fig. 3) in the following order: Tuotuo River, 0.271 t/t; Cuntan, 0.151 t/t; Yichang, 0.117 t/t; Wuhan Industrial Port, 0.127 t/t; Datong, 0.092 t/t; and Wusongkou, 0.091 t/t (Table 2). As CaO + MgO contents decreased, so did the TCS capacities. This result verified that CO_2_ was consumed by the dissolution of Ca–Mg minerals during sediment transport from upstream to downstream and that a carbon sink function existed. The TCS capacities at Cuntan and Wusongkou were 0.151 t/t and 0.091 t/t, respectively. A total of 0.060 tons of CO_2_ per ton of suspended sediment was dissolved during transport from Cuntan to the sea.

**Table 2:** The variation of carbon sink capacity and potential of suspended sediment along the Yangtze main stream.

Sampling point	Station	Basin area (10^4^ km^2^)	Average annual sediment flux (10^8^ tons) 1956–2000	Average CaO content of the three campaigns (%)	Average MgO content of the three campaigns (%)	TCS capacity (t/t) and potential (10^4^ tons)	Calcite content in July 2005 (%)	Dolomite content in July 2005 (%)	NSCS capacity(t/t) and potential (10^4^ tons)	SCS capacity (t/t) and potential (10^4^ tons)
M01	Tuotuo River	–	–	14.09	2.24	0.271	15.6	1.2	0.210	0.061
M07	Cuntan	86.7	4.39	4.92	3.35	0.151/6629	3.8	2.4	0.104/4566	0.047/2063
M12	Yichang	100.5	5.03	2.94	3.23	0.117/5885	0	4.1	0.078/3923	0.039/1962
M16	Wuhan Industrial Port	148.8	4.04	3.59	3.19	0.127/5131	4	3.4	0.097/3919	0.030/1212
M19	Datong	170.5	4.33	2.26	2.56	0.092/3984	1.8	2.4	0.065/2815	0.027/1169
M25	Wusongkou	–	–	2.18	2.56	0.091	0	1.5	0.051	0.040

The SCS capacities (C_3_) of silicate minerals in the sediment were in the range of 0.027–0.047 t/t and had little variation, except for the Tuotuo River (0.061 t/t). The NSCS capacity (C_2_) was consistent with the variation in the TCS capacity, and both had a gradual decreasing tendency from the headwater to the estuary (Fig. 3), as follows: Tuotuo River, 0.210 t/t; Cuntan, 0.104 t/t; Yichang, 0.078 t/t; Wuhan Industrial Port, 0.097 t/t; Datong, 0.065 t/t; and Wusongkou, 0.051 t/t. The silicate carbon sink capacity (SCS) was smaller and showed a smaller reduction than the NSCS along the Yangtze River, mainly due to the slower dissolution rate of silicate minerals or greater contribution of silicate rock clastics from the watersheds in the middle and lower reaches, which also limited CO_2_ consumption in comparison with carbonate minerals. Obviously, the decrease in TCS capacity from upstream to downstream was due to the intense dissolution of carbonate minerals in the Yangtze River.

An “ideal mainstream segment” refers to a segment where there was no sediment input from the tributaries or the amount of sediment supply from the tributaries was equal to the amount of sedimentation in the segment (meaning that the suspended sediment yields at the inlet and outlet were similar), and the calcium and magnesium mineral contents of suspended sediments in the tributaries and mainstream were similar. The carbon sinks via CO_2_ consumption by dissolution of calcium and magnesium minerals in the sediment transport process can be expressed as follows:

7 $${\text{Wt}}_{{{\text{h}}1 - 2}} = {\text{Ws}}_{{{\text{h}}1 - 2}} \times \left( {{\text{C}}_{{{\text{h}}1}}{-}{\text{C}}_{{{\text{h}}2}} } \right)$$

where Wth_1-2_ is the consumed CO_2_ via the dissolution of calcium and magnesium minerals in the segment (h_1_–h_2_) (10^4^ t/yr); W_Sh1-2_ is the mean suspended sediment transported at the segment (h_1_–h_2_) (10^4^ t/yr); C_h1_ is the carbon sink capacity of the suspended sediment at the inlet of the segment (t/t); and C_h2_ is the carbon sink capacity of the suspended sediment at the outlet of the segment (t/t).

The Yangtze River has a drainage area of 1785 × 10^6^ km^2^, in which the upper reaches above Yichang have an area of 1.05 × 10^6^ km^2^. The average sediment yield from 1956 to 2000 was 5.01 × 10^8^ t/yr at Yichang, while it was 4.33 × 10^8^ t/yr at Datong, with a drainage area of 1.705 × 10^6^ km^2^. In addition, it was 4.39 × 10^8^ t/yr at Cuntan^13^ with a drainage area of 0.867 × 10^6^ km^2^. Although there is a large drainage area of the river segment between Cuntan and Datong (0.838 × 10^6^ km^2^), the sediment yields at Cuntan and Datong were very similar, namely, 4.39 × 10^8^ t/yr, and 4.33 × 10^8^ t/yr, respectively, because sediment deposition in the channels of the segment offset the sediment supply from the tributaries. The contents of CaO and MgO in the suspended sediment in the tributaries (Hanjiang River, Ganjiang River and Xiangjiang River) in the middle and low reaches below the TGR dam were nearly the same as the values in the mainstream of the Yangtze River (Hankou station)^12^. Thus, we regarded the mainstream segment between Cuntan and Datong as an ideal mainstream segment, with Cuntan being upstream of the TGR dam, which made the evaluation of the damming effects available. The CaO + MgO contents of suspended sediment samples for the three campaigns (July 2003, July 2005 and July 2007) and the calcite + dolomite contents of the sediment (July 2005) at the two sites (Table 2) were used to calculate differences in TCS, NSCS and SCS capacities for the segment. Taking the mean sediment yield of the two sites during the period from 1956 to 2000 for reference, the annual net TCS, NSCS and SCS between the two sites were 26.45 × 10^6^ tons of CO_2_, 17.51 × 10^6^ tons of CO_2_ and 8.94 × 10^6^ tons of CO_2_, respectively. After 2001, due to hydropower exploration (especially the TGR project), ecological mitigation and soil conservation, the sediment yields at the two sites largely decreased and have stabilized since 2006. By comparison to the period before 2000, the sediment yields at Cuntan and Datong decreased by 72.4% and 71.6%, respectively, during the period of 2006–2019^14^. Due to the reduction in sediment yields, the annual net TCS, NSCS and SCS in the segment decreased by 18.52 × 10^6^ tons of CO_2_, 12.24 × 10^6^ tons of CO_2_ and 6.28 × 10^6^ tons of CO_2_, respectively (Table 3).

**Table 3:** Reduction in the carbon sink caused by the reduction in sediment transport in the reach between Cuntan Station and Datong Station after 2006.

Station	Average annual sediment flux (10^8^ tons/yr)		Carbon sink reduction (10^4^ tons of CO_2_/yr)		
	1956–2000	2006–2019	Total carbon sink	Nonsubstantial	Substantial
Cuntan	4.39	1.21	1852	1224	628
Datong	4.33	1.23			

### Carbon sink capacities of the global rivers and their implications

The amount of deposited sediments of the Three Gorges Reservoir (TGR) from June 2003 to December 2017 was 1.669 × 10^9^ tons, and the average sedimentation rate was 114.5 × 10^6^ t/yr^15^. According to the differences in the TCS, NSCS and SCS capacities of the suspended sediments between Cuntan and Datong, the losses of annual TCS, NSCS and SCS by sedimentation in the TGR were estimated to be approximately 6.756 × 10^6^ tons of CO_2_, 4.466 × 10^6^ tons of CO_2_ and 2.290 × 10^6^ tons of CO_2_ × 10^6^ tons of CO_2_, respectively. The power generation of the Three Gorges Hydropower Station exceeded 100 × 10^9^ kW/h in 2018, equivalent to saving 31.9 × 10^6^ tons of standard coal and reducing 85.80 × 10^6^ tons of CO_2_ emissions^16^. The reduction in inorganic carbon sinks from the sedimentation in the TGR was equivalent only to a limited amount (TCS for 7.9% and SCS for 2.7%) of the reduced CO_2_ emissions by the Three Gorges Hydropower Station. Moreover, the sediment deposited by the TGR could bury and store vast quantities of organic carbon^6^, and particulate organic carbon (POC) contents in the TGR in recent reports^17–19^ varied from 0.26 to 9.2%. An average of 1.5% of POC in buried sediment was used to estimate annual buried organic carbon, and the relatively permanent sedimentation of organic carbon was equivalent to 6.30 × 10^6^ tons of CO_2_ sequestration. The losses of annual TCS and SCS via silicate weathering by the TGR project could also offset 107.28% and 36.36% of its annual CO_2_ sequestration (6.30 × 10^6^ tons) via permanent sedimentation, respectively.

From a global perspective, 4462 rivers with basin areas of more than 100 km^2^ showed that the current annual sediment flux of global rivers into the sea was 12.61 × 10^9^ tons^20^. Taking the preimpoundment period TCS of 0.060 t/t for the stream segment between Cuntan and Wusongkou (the mouth of the Yangtze River) into consideration, the total inorganic carbon sink amount of 7.57 × 10^8^ tons of CO_2_ was derived from global rivers, which is equivalent to 71.6% of the total inorganic carbon sink of global rock weathering (1.056 × 10^9^ tons of CO_2_), with weathered silicate being more than 26% of the total weathered rocks^4,6,10^. The enhanced silicate rock weathering (ERW) strategy proposed by Beerling et al.^1^ would create a higher annual SCS, reaching 2 × 10^9^ tons of sequestered CO_2_. To achieve this goal, there is no doubt that the inorganic carbon sink amount contributed by the dissolution of calcium and magnesium minerals in the processes of river sediment transport accounted for a great portion of the carbon sink amount via global rock weathering. The collision and abrasion of river sediments combined with stirring and mixing could promote the dissolution of minerals during sediment transport processes (off-site weathering of the rock). Therefore, it was suggested that the dissolution rate of off-site rock weathering was higher than that of in situ weathering. In comparison to the periods with limited anthropogenic influences, the global sediment fluxes to the sea decreased by approximately 10%^20^, and the corresponding total carbon sink loss in a year was estimated to be 0.757 × 10^9^ tons of CO_2_, which was still less than the amount of CO_2_ emission reduction contributed by hydropower exploration and the associated buried organic carbon per year.

## Conclusions

The rock weathering processes included in situ weathering during the development of weathering crust and off-site weathering during sediment transport. The dissolution of silicate and carbonate minerals in river sediment consumed CO_2_ in river water. From June 2003 to December 2017, the average annual sedimentation of the Three Gorges Reservoir (TGR) was 114.5 × 10^6^ tons, and the related reduction in inorganic carbon sinks from sedimentation by the TGR was only equivalent to a limited amount (7.9% for the TCS and 2.7% for the SCS) of the reduced CO_2_ emissions by the Three Gorges Hydropower Station. The TCS of global rivers was estimated as 757 × 10^6^ tons, which is equivalent to 71.6% of the TCS by global rock weathering with 1.06 × 10^9^ tons of sequestered CO_2_, among which the SCS was more than one quarter of the TCS. The dissolution of calcium and magnesium minerals during river sediment transport accounted for a great portion of the carbon sink amount by global rock weathering. The collision and abrasion of river sediments combined with stirring and mixing could promote the dissolution of minerals during sediment transport processes (off-site weathering of the rock). Therefore, it is suggested that more attention should be given to dissolution processes and rates of off-site rock weathering, which might be more significant than in situ weathering, especially in the upper reaches of large rivers.
